# Fipronil induces lung inflammation in vivo and cell death in vitro

**DOI:** 10.1186/s12995-016-0102-0

**Published:** 2016-03-18

**Authors:** Kaitlin Merkowsky, Ram S. Sethi, Jatinder P. S. Gill, Baljit Singh

**Affiliations:** Department of Veterinary Biomedical Sciences, Western College of Veterinary Medicine, University of Saskatchewan, 52 Campus Drive, Saskatoon, SK S7N 5B4 Canada; School of Animal Biotechnology, Guru Angad Dev Veterinary and Animal Sciences University, Ludhiana, India; School of Veterinary Public Health, Guru Angad Dev Veterinary and Animal Sciences University, Ludhiana, India

**Keywords:** Macrophage, Pesticides, Lung Inflammation, TLR4, TLR9

## Abstract

**Background:**

Fipronil is an insecticide that acts at the *gamma*-aminobutyric acid receptor and glutamate-gated chloride channels in the central nervous systems of target organisms. The use of fipronil is increasing across the globe. Presently, very little data exist on the potential impact of exposure to fipronil on the lungs.

**Methods:**

We studied effects of intranasal (*N* = 8) and oral (*N* = 8) treatment with fipronil (10 mg/kg) on lungs of mice. Control mice were given groundnut oil orally (*N* = 7) or ethanol intranasally (*N* = 7) as these were the vehicles for respective treatments.

**Results:**

Hematoxylin-eosin stained lung sections showed normal histology in the control lungs compared to the thickened alveolar septa, disruption of the airways epithelium and damage to vascular endothelium in the intranasal and the oral groups. Mice exposed to fipronil either orally or intranasally showed increased von Willebrand factor staining in the endothelium and septal capillaries. Compared to the control mice, TLR4 expression in airway epithelium was increased in mice treated intranasally but not orally with fipronil. Oral fipronil reduced TLR9 staining in the airway epithelium but intranasal exposure caused intense staining in the alveolar septa and airway epithelium. There were higher numbers of TLR4 positive cells in alveolar septa in lungs of mice treated intranasally (*P* = 0.010) compared to the respective control and orally treated mice but no significant differences between treatments for TLR9 positive stained cells (*P* = 0.226). The U937 macrophage cells exposed to fipronil at concentrations of 0.29 μm to 5.72 μm/ml over 3- or 24-hour showed significant increase in cell death at higher concentrations of fipronil (*P* < 0.0001). Western blots revealed no effect of fipronil on TLR4 (*P* = 0.49) or TLR9 (*P* = 0.94) expression on macrophage cell line.

**Conclusion:**

While both oral or intranasal fipronil treatments induced signs of lung inflammation, the number TLR4-positive septal cells was increased only following intranasal treatment. Fipronil causes macrophage cell death without altering TLR4 and TLR9 expression in vitro.

## Background

The Sumerians were probably the first to used elemental sulphur against insects and mites in 2500 BC and ancient Chinese cultures treated body lice with arsenic and mercury. While the ingenuity of these ancient cultures can be appreciated, it is unlikely arsenic would be embraced today as an effective pesticide due to its deleterious effects on human health. The most prominent example in modern times of the struggle between insect control and public health concerns is dichlorodiphenyltrichloroethane (DDT), which belongs to organochlorine group of pesticides [1], and showed unparalleled effectiveness in bringing down cases of malaria. While pesticides and insecticides are the tools to manage pests and insects, many health effects of these chemicals have also be recorded.

Fipronil ((±)-5-amino-1- (2,6-dichloro-α,α,α-trifluoro-p-tolyl)- 4-trifluoromethylsulfinylpyrazole-3-carbonitrile) belongs to the phenylpyrazole family and is extensively used around the world in various anti-flea and tick sprays and for pest control in agriculture [2]. Fipronil acts as an antagonist at GABA-gated chloride channels but has a higher affinity for these channels in insects compared to non-target organisms such as humans, making it a seemingly safer product in these regards [3–5]. Fipronil is metabolized into many metabolites including sulfone, sulphide, and desulfinyl. However, it has been demonstrated the primary metabolite of fipronil, fipronil sulfone, actually has a much greater affinity for these channels in mammals than those in insects indicating potential detrimental effects of the break-down products to non-target organisms [6]. One of the studies found that following a single oral exposure fipronil sulfone persists for much longer duration than fipronil in high fat containing tissues especially adipose tissue, adrenals and the liver which leads to bioamplification along the food chain [7, 8]. It has been shown that both sulfone and desulfinyl are potent mitochondrial uncouplers and calcium efflux inducers but may differ in their potencies [9]. Nevertheless, exposure to the parent compound is pre-requisite for the generation of fipronil metabolites. Combined subchronic exposure to fipronil and fluoride induces biochemical alterations in buffalo calves [10, 11]. Fipronil also induces oxidative stress and activation of MAPK, induction of apoptosis via caspase-9 and caspase-3, and reduction in differentiated cell numbers [12–14]. During an examination of effects of fipronil on liver p450 enzymes, it was found that fipronil is an inducer of hepatic phase I CYP enzymes that may increase potential for interactions with xenobiotics [15]. While recent studies have shown the toxic effects of fipronil on liver especially the mitochondria in liver cells [9, 16], there currently are no data on the pulmonary effects of fipronil in animals.

This study was designed to investigate the pulmonary effects of fipronil following oral or intranasal exposures through testing of hypothesis that exposure to low levels of fipronil will induce lung inflammation and cell death. The inhalation route remains a major route of exposure in developing countries where agricultural workers generally spray pesticides without appropriate personal protective gear. The oral route occurs through contamination of food and water with pesticides. We also investigated the effects of fipronil on a macrophage cell line in vitro*.* The data show fipronil causes cell death in vitro and induces lung inflammation following both oral and intranasal routes of administration but increases number of TLR4 cells only after intranasal treatment.

## Methods

### In vivo experiments

Experiment was conducted following approval from the Institutional Animal Ethics Committee (IAEC), Guru Angad Dev Veterinary and Animal Sciences University, Ludhiana. Swiss albino mice, ages 8–10 weeks, were housed in laboratory animal cages at 18–22 °C and 12:12 light–dark cycles. Mice had access to feed (Ashirwad Industries, Chandigarh, Punjab, India) and water ad libitum.

### Experiment design

An initial experiment was first conducted where mice (*n* = 8) were exposed to 8 mg/kg or 2 mg/kg of fipronil via intranasal or oral routes (*n* = 2 each) or treated with respective vehicles (*n* = 2 each for ethanol for intransal and corn oil for oral route) to determine an appropriate dose of fipronil (Sigma-Aldrich S. Louis, USA; <=100 % purity). Based on the preliminary study, we decided on 8 mg/kg of fipronil as the dose for both intranasal and oral exposure routes. This dose was determined as it was 10 % of the oral LD_50_ for mice to reduce chances of acute toxicity and death and treated mice for seven days to induce sub-chronic toxic effects (http://npic.orst.edu/factsheets/fiptech.pdf).

Control mice (*N* = 7) received groundnut oil, which was used as a vehicle for treating mice (*N* = 8) orally with fipronil (8 mg/kg/day). Mice (*N* = 8) were treated intranasally with fipronil (8 mg/kg/day) in dissolved in ethanol. Control mice (*N* = 7) received intranasal treatment ethanol. Unfortunately the intranasal groups of mice experienced high mortality following anaesthesia and the ketamine/xylazine dose was adjusted daily to reduce chances of mortality in the mice.

After 7 days mice were euthanized with a lethal dose of ketamine/xylazine (0.1 μl/10 g of body weight) and cardiac puncture was done to collect blood. The trachea was isolated and a small cannula was inserted to perform lung lavage. Lungs were lavaged 3 times with 0.5 ml of phosphate buffered saline to collect broncho-alveolar lavage (BAL) fluid. The left lung was fixed in 10 % formalin overnight (24 h). Lungs were placed in filter capsules and processed by the acetone and benzene method to obtain 5 μm thick paraffin sections.

### Hematoxylin and eosin staining

The paraffin sections of lungs from all groups were stained with H&E staining for routine histopathology.

### Immunohistochemistry

The immunohistochemistry was performed as reported earlier (Sethi 2013). Briefly tissue sections were de-paraffinized and rehydrated. The tissue peroxidases were inactivated with 0.5 % H_2_O_2_ in methanol for 20 min. Pepsin (2 mg/ml in 0.01 N HCl) was used to unmask antigen-binding sites (60 min) and then 1 % bovine serum albumin (BSA) in PBS was used to prevent non-specific binding (30 min). Next the tissues were incubated overnight (16 h) at 4 °C with the following antibodies: von Willebrand Factor (1:500, DAKO A0082), Toll-Like Receptor 4 (1:25, IMG-578A, IMGENEX) and Toll-Like Receptor 9 (1:50, IMG-3051, IMGENEX) followed by appropriate secondary antibody (vWF at 1:300 and TLR at 1:100, all from DAKO). VECTOR VIP Peroxidase Substrate Kit (Vector laboratories, Burlingame, CA) was used for colour-development ed followed by counter staining with methyl green (Vector laboratories).

### Grading for immunohistochemistry

Lungs sample from all the animals were used for grading of immunohistochemical staining intensity and quantification of the number of TLR4 or TLR9 positive cells in the alveolar septa (Table B). For each tissue section five random fields of vision was assessed and a score of 1–4 (1 being least intense, 4 being most intense) was assigned for each of the criteria. The cells were counted in 5 consecutive fields under 100X. The sections were graded depending on the staining intensity of vWF in the large blood vessels, or TLR4 or TLR9 in the bronchial epithelium and continuity of the airways epithelium as interrupted or intact (Table B). One individual who was blinded to what treatments of each animal performed the scoring.

### In vitro experiments

U937 cell line was obtained from ATCC®. RPMI Complete Media +10 % Fetal Bovine Serum (FBS) (Gibco) was warmed to 37 °C. Aliquots of cells were thawed in a hot water bath (37 °C) and combined with 10 ml warm media in 15 ml clinical centrifuge tubes. The tubes were spun at 258 *g* for 3 min, the supernatant was discarded and the pellet then resuspended in 10 ml media in a closed flask and incubated at 37 °C, 5 % CO_2_. The cell suspension was removed from the flask and transferred to a 15 ml centrifuge tube and spun at 258 *g* for 3 min. The supernatant was discarded and cells were resuspended in 5 ml media, split and transferred into 2 new flasks and topped with media (~15 ml). Incubation continued at 37 °C, 5 % CO_2_, and this procedure was repeated every 2–3 days till the desired concentration (5 × 10^6^ cells/ml) of cells was achieved.

### Fipronil exposure and viability assessment

Once the cells reached a concentration of 5 × 10^5^ cells/ml, the cells were incubated with 12 ml media + Phorbol-12-myristate-13-acetate (PMA) in 12 well plate for 48 h to differentiate into macrophages. The cells were treated with various concentrations of fipronil (0.29 μm to 5.72 μm per 1 ml) dissolved in DMSO for 3 h. Following desired incubation, 50 μl of 0.25 % Trypsin in Ethylenediaminetetraacetic acid (EDTA) was added to each well in order to facilitate removal of the differentiated macrophages. 150 μl of the cells in media were combined with 50 μl Trypan blue to assess the viability of cells by trypan blue exclusion method.

### Western blots for TLR4 and TLR9

Following a 3-hour exposure to fipronil, 50 μl of 0.25 % Trypsin in EDTA was added to each well for 5 min. Each sample was collected in 1.5 ml centrifuge tubes and spun for 10 min at 258 *g*. The supernatant was aspirated off and the pellet was washed with 1 ml Hanks Balanced Salt Solution (HBSS). This step was repeated twice. The supernatant was again aspirated off and one tablet of Protease inhibitor (Roche) was added to 7 ml Radioimmunoprecipitation assay (RIPA) (Sigma-Aldrich) buffer. A 300 μl of this RIPA buffer mix was added to each centrifuge tube and vortexed thoroughly. The samples were kept in a 4 °C refrigerator for 15 min and vortexed twice during this incubation. The samples were put in the centrifuge at 10,000 *g* for 5 min. The supernatant was then carefully removed without disturbing the pellet and stored at −45 °C until needed for future assays for up to 1 month. 25 μl of protein samples plus indicator were boiled for 5 min and then loaded into a 12 % SDS-PAGE gel. The proteins were separated via gel electrophoresis at 160 V for 45−60 min. The gel was collected and placed between 2 sponges, 4 filter papers and Immobilon – FL membrane in a western blot sandwich all previously soaked in the transfer buffer. The protein transfer was performed at 100 V for 70 min. Following transfer, the membrane was washed in 15 ml PBS and then incubated in ~15 ml blocking buffer (5 % BSA in PBS) for 1 h. Primary antibodies were mixed with the above blocking buffer with the addition of 0.1 % Tween-20. Primary antibodies (Anti-TLR4, AF1478 R&D at 1:200 and Anti-TLR9, IMG 305A at 1:200, IMGENEX) were incubated with membranes overnight at 4 °C. Following overnight incubation membranes were washed with PBS and PBST and then incubated with secondary antibody (Goat, anti-mouse Cy5.5 or Donkey Anti-goat Cy3, both at 1:1000, AbCam) in PBS for 30 min. Washing was repeated after this step and then membranes were allowed to dry before visualization using the Typhoon 3 laser fluorescence scanner.

### Statistical analyses

Statistical analysis was performed using statistical software (SPSS, IBM version 21.1 for Windows). For in vivo work a one-way analysis of variance (ANOVA) was run to determine if the number of TLR4/9 positive cells present in the alveolar septa was significantly different between treatments groups. If there was a significant difference, Tukey’s Multiple Comparison test was performed to see which treatments differed. For in vitro work, one-way or two-way ANOVA was run to see if there were significant difference between average cell viability. Dunnet’s or Tukey’s Multiple Comparison Test was ran to see the differences. Since only 2 treatments were used for western blots, Student’s Independent *T*-test was used to determine significant differences.

## Results

### Hematoxylin and eosin staining

**Fig. 1:**
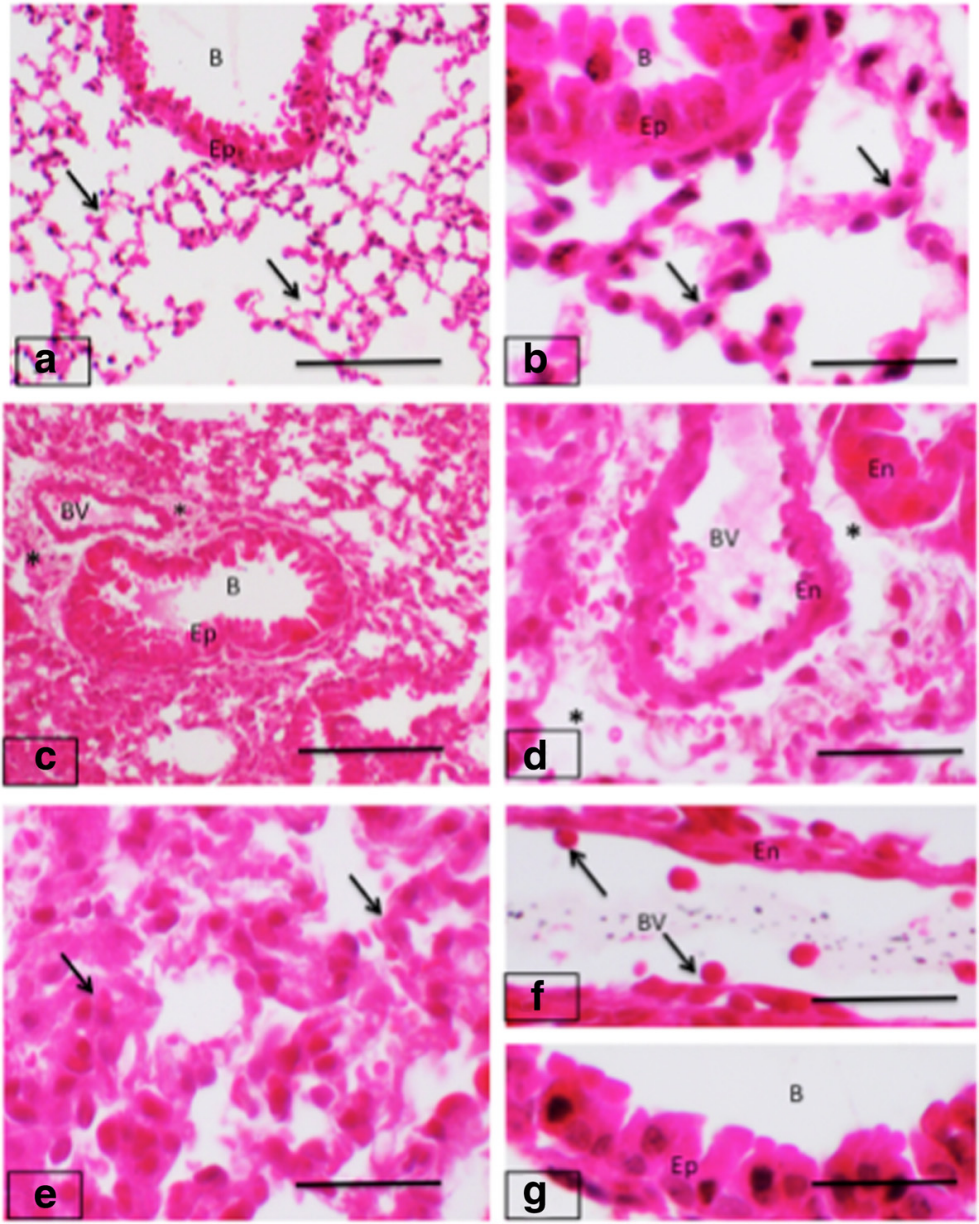
H&E staining of mice lungs. Lung sections from control mice (**a-b**) have normal lung histology of alveolar septa *(arrows*) and bronchiolar (B) epithelium (Ep). The oral treatment with fipronil caused lung inflammation and lung sections (**c-d**) show inflammation (*asterisks*) around bronchioles (B) and blood vessels (BV). Lung sections from mice treated intranasally with fipronil (**e-g**) show septal congestion in septa (*arrows*; **e**)), cells adhering (*arrows*) to endothelium (En; **f**) and swollen epithelium (Ep) of bronchioles (B; 1 g). Bar: 100 μm

H&E stained lung sections from control group showed normal histoarchitecture of lungs except few black spots indicating some evidence of dust particles that the animals could have easily been exposed to throughout the experiment (Fig. 1a and b). Lung sections from animals in the oral fipronil group displayed an accumulation of inflammatory cells around the terminal bronchioles. There was a dilatation of perivascular spaces in lung sections from all the animals. The airway epithelial cells were enlarged and domed in mice treated intranasally with fipronil (Fig. 1c and d). The blood vessel showed a folded appearance. In comparison to the oral group, the intranasal fipronil group displayed overall normal lung architecture and the epithelium did not appear to be activated but there was an increase in accumulation of inflammatory cells in the alveolar septa and the alveoli (Fig. 1e, f and g). Many blood cells were attached to the vascular endothelium (Fig. 1f).

### Expression of von Willebrand factor

**Fig. 2:**
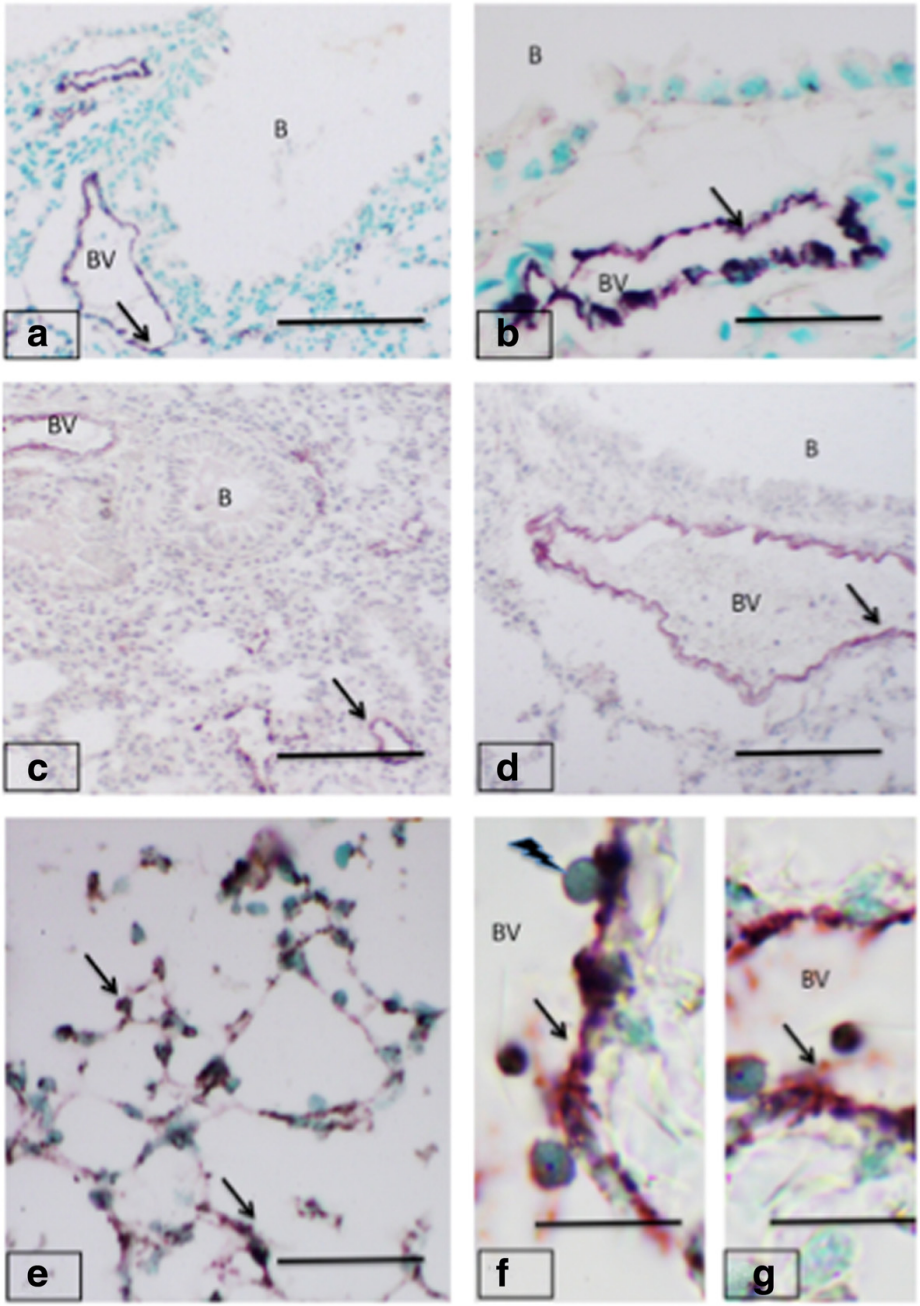
von Willebrand Factor expression in mice lungs. Lungs from control groups (**a-b**) show normal vWF staining (*arrows*) in endothelium of blood vessels (BV) but not in bronchiolar (B) epithelium. The oral treatment with fipronil (**c-d**) caused inflammation but the expression of vWF (*arrows*) in blood vessels (BV) remained unchanged. The intranasal fipronil (**e-g**) showed increased expression (*arrows*) in alveolar septum (**e**), endothelium of blood vessels (**f-g**) as well as the vascular cells. Note vascular cells (*lightening arrow*) attaching to the endothelium (**f**). Bar: 100 μm

Lung sections from control animals showed immunopositive staining for vWF in vascular endothelium that was more prominent in larger blood vessels compared to the alveolar septal capillaries (Fig. 2a and b). There was no vWF staining of the bronchiolar epithelium in lung sections from any of the treatment groups. The mice of the oral fipronil group did not have an altered expression of vWF though there was an indication of inflammation in the lung (Fig. 2c and d). An increase in vWF staining was displayed in lung sections from the intranasal fipronil group in areas such as septal capillaries and in the cells accumulated in septal areas (Fig. 2e, f and g). There was specially increased focal vWF staining in endothelial cells and the adhering blood cells (Fig. 2f).

### Expression of Toll-like Receptor 4

**Fig. 3:**
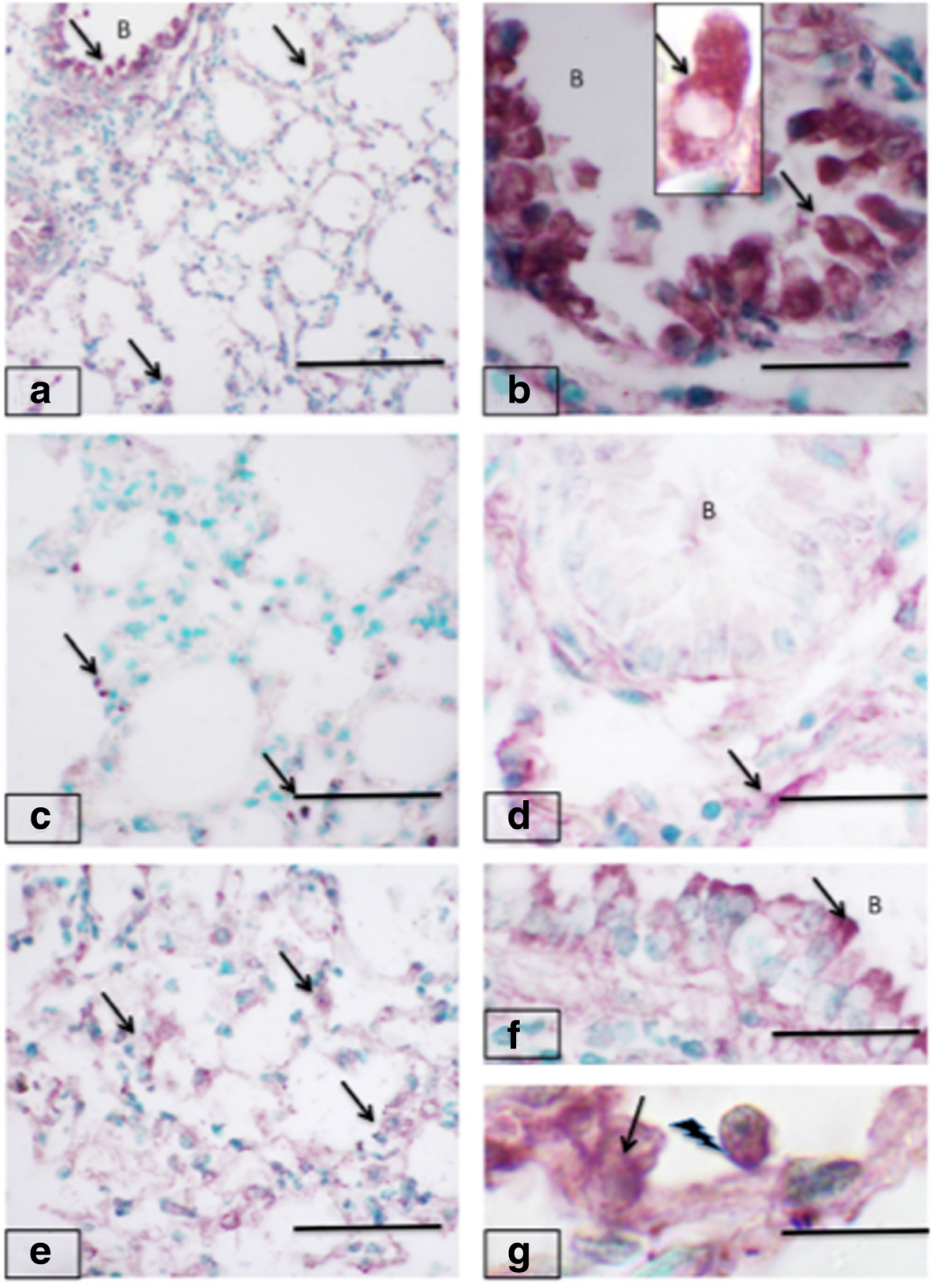
Toll-like receptor 4 expression in mice lungs. Lungs from control groups (**a-b** and) show strong staining (*arrows*) in bronchiolar epithelium (B) and alveolar septum. Note rich cytoplasmic staining in bronchiolar epithelial cells (**b** and inset). The lungs sections (**c-d**) from mice treated orally with fipronil show barely minimal staining (*arrows*) in alveolar septum (**c**) while it is nearly absent in bronchiolar (B) epithelium. Lung sections from mice exposed to fipronil (**e-g**) show TLR4 staining similar (*arrows*) to the control lungs in alveolar septum (**e**) and epithelium (*arrows*) of bronchioles (B, **f**). Note TLR4 staining (3 g) in endothelium (*arrow*) and adhering cells (*lightening arrows*). Bar = 100 μm

The immunopositive TLR4 cells were observed in the cytoplasm of bronchiolar epithelium of oral and intranasal control groups (Fig. 3a and b). Bronchial associated lymphoid tissues (BALT) were present, however indicating the animals were kept in a dusty environment, as BALTs are not normally present in animals kept in sterile environments. The BALTs lacked TLR4 staining. There was reduced staining for TLR4 in lungs of mice administered fipronil orally (Fig. 3c, d). The intranasal fipronil group showed TLR4 staining in alveolar septa, bronchiolar epithelium and vascular endothelium (Fig. 3e, f, g). There reaction was more marked in the apical surface of the airway epithelium (Fig. 3f). The interface of blood cells, vascular endothelium and alveolar macrophages also showed TLR4 staining (Fig. 3g). There was significantly higher number of TLR4 positive cells in the alveolar septa in the intranasal fipronil group compared to intranasal control (*P* = 0.050) and oral fipronil group (*P* = 0.010).

### Expression of Toll-like Receptor 9

**Fig. 4:**
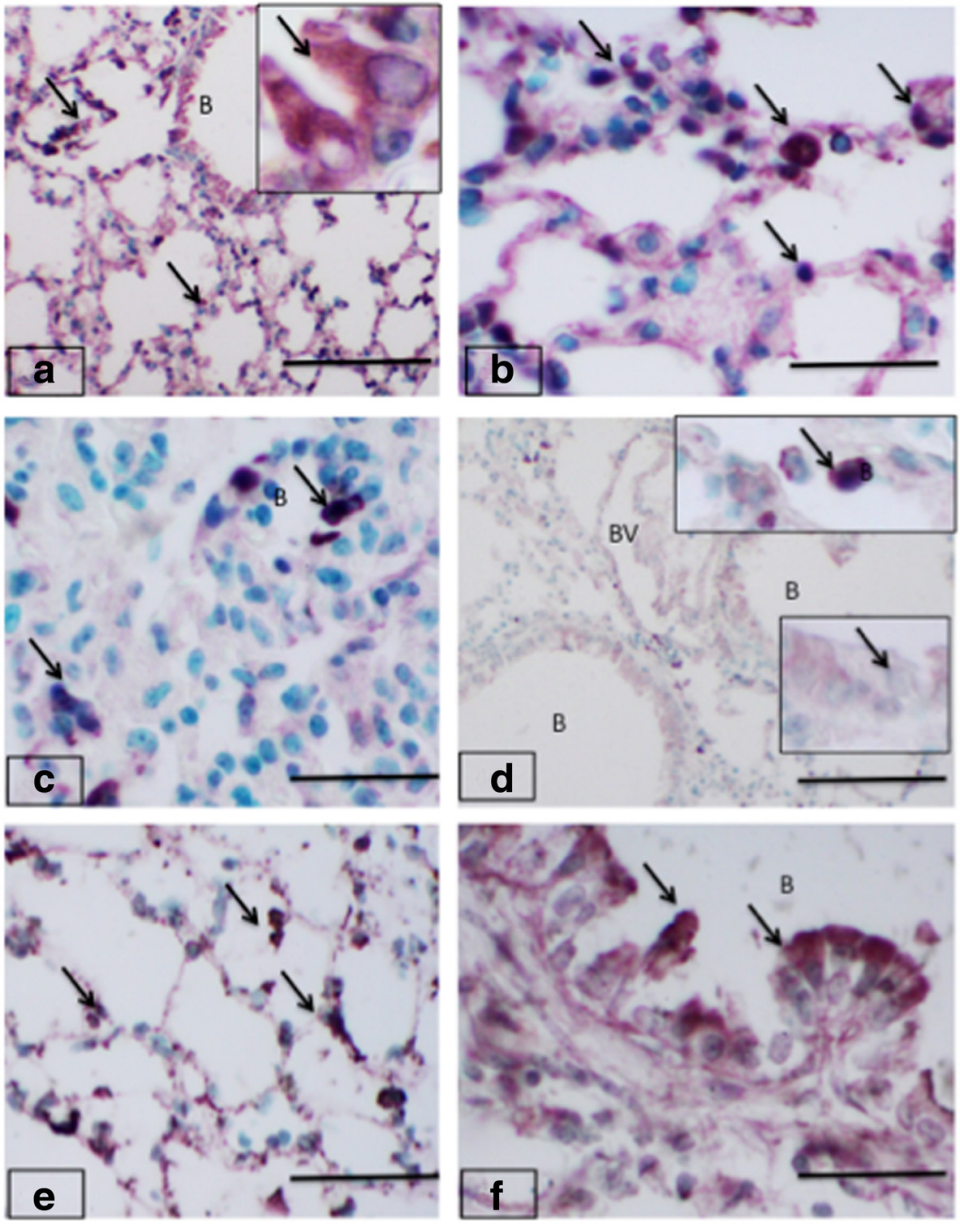
Toll-like receptor 9 expression in mice lungs. Lungs sections from control groups (**a-b**) have TLR9 staining (*arrows*) in alveolar septum (**a**) and bronchiolar epithelium (**a** inset). The staining is also seen in the septal cells (*arrows*) in lung sections from control mice (**b**). The oral treatment reduced TLR9 staining in lung sections (**c-d**) and the staining (*arrows*) was observed in occasional septal cells (**c**). Bronchiolar epithelium (B; **d** and insets) showed much reduced TLR9 staining compared to the controls. Lung sections from mice treated with intranasal fipronil (**e-f**) showed intense staining (*arrows*) in alveolar septum (**e**) and bronchiolar epithelium (**f**). Bar: 100 μm

TLR9 staining was observed in septa, airway epithelium and blood vessels in the lungs sectins from the oral and intranasal control groups (Fig. 4a, b). Further,, TLR9 staining assumed focal appearance in the septa but the airway epithelial staining was considerably reduced in oral fipronil group (Fig. 4c, d). There was minimal TLR9 reaction in vascular endothelium (Fig. 4d). However, TLR9 expression was intense and more prominent in the alveolar septa specially in the large cells in lungs of mice exposed intranasally to fipronil (Fig. 4e). The airway epithelium also showed intense surface staining for TLR9 while the cytoplasmic reaction was reduced in this group compared to control group (Fig. 4f). There was no significant difference for the number of TLR9 positive cells among treatment and control groups.

### In vitro results

#### Fipronil reduces cell viability

**Fig. 5:**
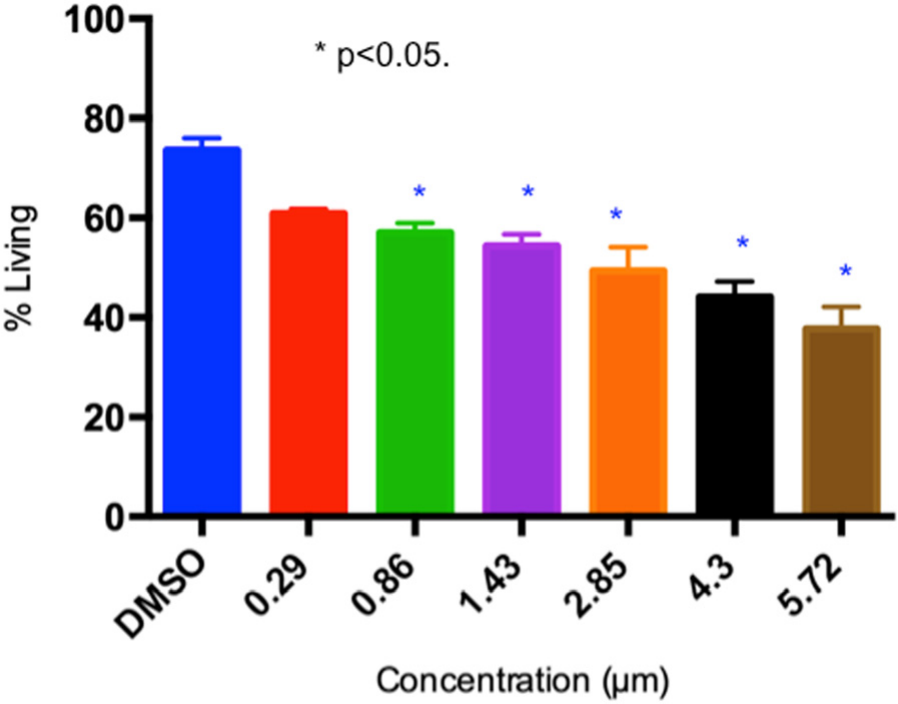
Average U937 cell viability (%) is presented as a function of concentration (μm) of fipronil. Data are presented as means with error bars representing standard error of the mean. Significant results were obtained with a 1-way analysis of variance (ANOVA) and Dunnet’s Multiple comparison Test (*F*_3,13_ = 1.651, *P* = 0.226). * depicts the result is significantly different from the control (DMSO). *F*_6,67_ = 14.03, *P* < 0.0001)

**Fig. 6:**
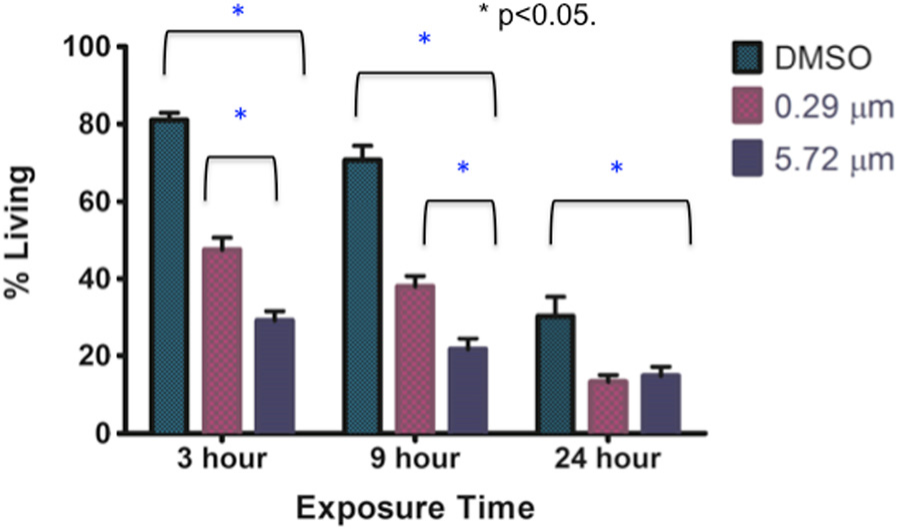
Average cell viability (%) as a function of concentration (μm). Data are presented as means with error bars representing standard error of the mean. Significant results were obtained via a 2-way ANOVA and Tukey’s Multiple Comparison Test. No significant result was found for an interaction between time and concentration (*F*_4,120_ = 11.01, *P* = 0.115).) There was a signicant result for time (*F*_2,120_ = 29.6, *P* <0.0001) and for concentration (*F*_2,120_ = 94.82, *P* <0.001). * represents significant results

There was a significant difference in percentage of living cells between the control and both fipronil concentrations. At 3 and 9 h the low fipronil concentration also had a significantly higher percentage of living cells than the high concentration (Fig. 5). At 24 h there was not a significant difference in the % of living cells between the low and high fipronil concentrations (Fig. 6). Further, there was no significant interaction between time vs. concentration (*F*_4,120_ = 11.01, P = 0.115).

#### Expression of TLR4 and TLR9

**Fig. 7:**
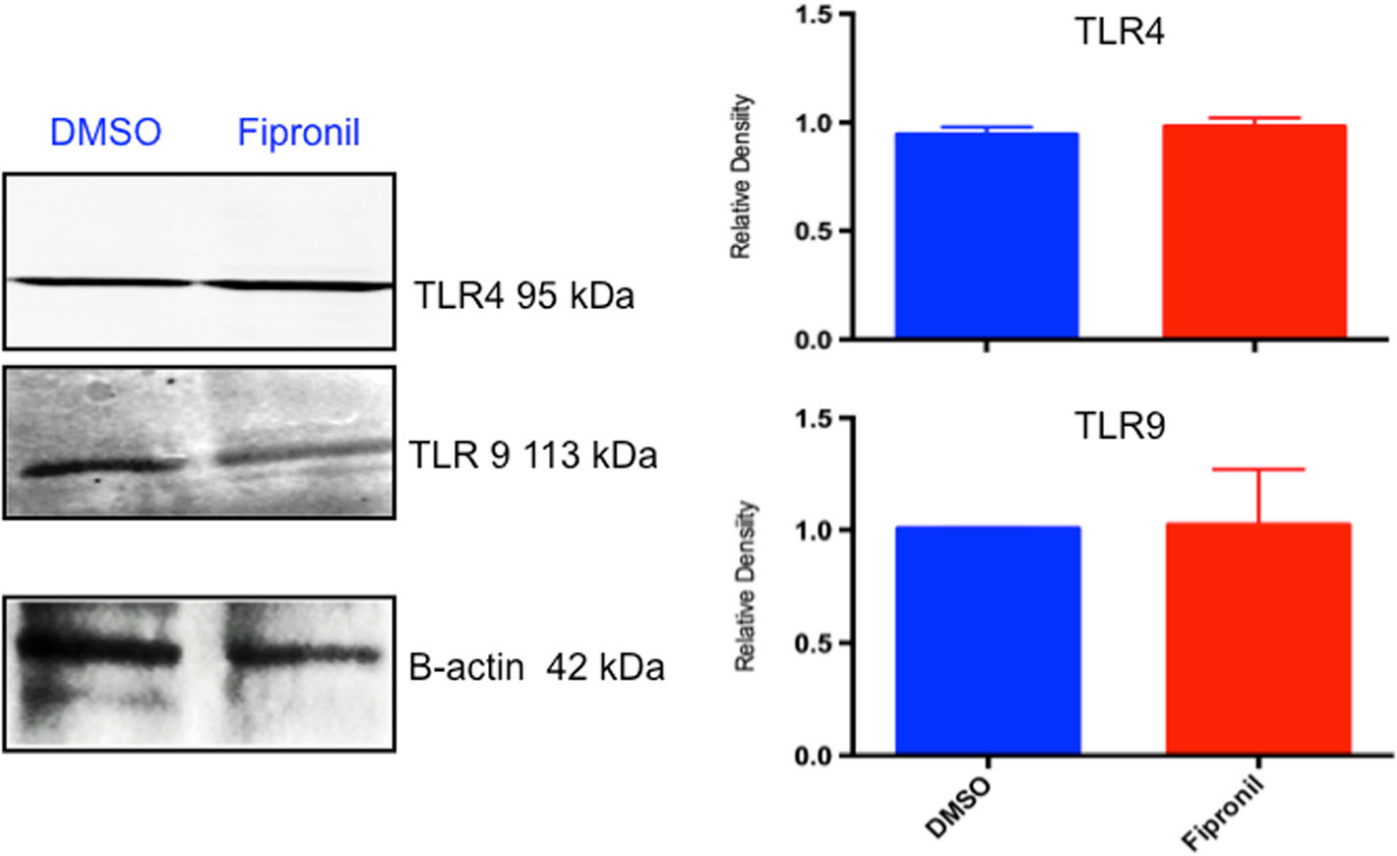
Western blot of TLR4 and TLR9: Western blots show TLR4 expression cells treated with fipronil and DMSO (5.72 μm). The experiment was repeated three times. Densitometry didn’t reveal any differences in the expression of TLR4 (*P* = 0.49) and TLR9 (*P* = 0.94)

After the U937 cells were incubated with a high (5.72 μm) concentration of fipronil for 3 h, protein extraction was done and western blots were performed for TLR4 and TLR9 followed by densitometric quantification. Figure 7 depicts the average relative density of western blots that were performed three times. The expression of TLR4 and TLR9 did not show any significant difference between the group treated with fipronil vs. the group treated only with DMSO. TLR4 (*P* = 0.49), TLR9 (*P* = 0.94).

## Discussion and conclusions

We report the first in vivo and in vitro effects on lungs and macrophage cells, respectively, of fipronil. The data show lung inflammation following both oral and intranasal treatments with fipronil and an increase in TLR4 positive cells in alveolar septa with intranasal treatment. The in vitro treatment with fipronil caused a concentration dependent reduction in the number of viable U937 macrophage cells but had no effect on the TLR4 or TLR9 expression. Taken together, these data suggest that exposure to fipronil induces lung inflammation and may increase its susceptibility for subsequent endotoxin exposure.

Because of the growing use of fipronil as a pesticide, we examined the effects of oral and intranasal exposures to fipronil on non target organs like lungs. Because this was the first study on pulmonary effects of fipronil, we chose to use fipronil and to focus on any of its metabolite such as sulfone or disulfiny in later studies. Fipronil resulted in lung inflammation in mice as evidenced by accumulation of inflammatory cells, which is considered a hallmark of inflammation [17]. Further, In addition to the routine histology, we also used vWF as a marker of inflammation. the expression of vWF was especially upregaulated in the septal capillaries in mice treated intranasally with fipronil suggesting signs of microvascular inflammation. The expression of vWF in lungs of mice treated orally with fipronil remained changed except some apparent increase in larger pulmonary blood vessels. vWF is a resident adhesive protein in Wiebel-Palade bodies in endothelial cells, which is exocytosed along with IL-8 and P-selectin by activated endothelial cells [18, 19]. The data suggest that fipronil given orally or intranasally induced lung inflammation including that in the vasculature.

Lung inflammation is regulated through activation of innate immune system comprised of Toll-like receptors such as TLR4 and TLR9 that bind, respectively, to lipopolysaccharides and CpG molecules [20–22]. We report the first study that fipronil given orally apparently reduced the immunohistochemical expression of TLR4 and TLR9. Intranasal treatment with fipronil increased airway epithelial and vascular endothleial expression of TLR4 and TLR9. The intranasal treatment also increased the number of septal cells expressing TLR4. Previously, the herbicide paraquat given intraperitoneally increased in TLR4 mRNA in the myocardium [23]. Oral treatment with sodium methyldithiocarbamate (SMD), a commonly used pesticide in the U.S., altered expression of TLR4 and inhibited the MAP kinases, which are down-stream of TLR4, to reduce the production of pro-inflammatory cytokines [24, 25]. Therefore, the observed increase in TLR4 expressing cells in the alveolar septa would increase susceptibility of animal and humans to endotoxin exposures, which are found in higher amounts in agriculture buildings such as grain elevators and pig barns. Therefore, we need to undertake additional studies where experimental animals exposed to fipronil are challenged with LPS to understand the interactions between the two. The present studies also do not address the reasons for a significant increase in TLR4 positive cells after intranasal but not oral treatment with fipronil. One of the reasons may be the fipronil in the oral group was metabolized and a significantly lesser amount of the original chemical or the metabolite reached the lungs thus activating a fewer number of TLR4 positive cells. Possibly the passage of the chemical or its metabolite(s) through the liver may have attenuated its toxicity for the lung.

We used a macrophage cell line for in vitro studies on the effects of fipronil as many of the septal cells recruited following intranasal treatment are macrophages. Macrophages are central to lung immunity and generation of immune responses [26]. The data showed a reduction in percentages of living cells with a range of concentrations from 0.86 μmol to 5.72 μmol but not at 0.29 μmol. We used 0.29 μmol and 5.72 μmol and found that only the higher concentration caused significant reduction in cell viability at 3 and 9 h of incubation with fipronil compared to the control and the lower concentration. However, both concentrations reduced cell viability after 24 h of the exposure. Previously, chlorpyrifos, which is linked to abnormal immune responses in humans induced apoptosis in U937 cells through caspase-3 activation [27]. Our histological observations didn’t reveal signs of cell necrosis/apoptosis in lungs following in vivo treatment with fipronil. The in vitro induction of cell death by fipronil may be due to more direct interaction of the pesticide with only one cell type available in the culture, and requires additional studies.

Macrophages express TLR such as TLR4 and TLR9 and use these molecules to sense microbial molecules [26, 28]. Western blot data showed lack of effect of fipronil on TLR4 and TLR9 expression in macrophage cell line. Because fipronil kills macrophages, the lack of differences in TLR4 and TLR9 expression may be due to increased expression of TLR4 and TLR9/cell to result in lack of differences between the control and the treated macrophage. This would in turn suggest that the expression of TLR4 and TLR9 on live cells may actually be increased as was the case in vivo where the number of septal cells expressing TLR4 was increased following intranasal treatment with fipronil. The preliminary immunofluorescence data on TLR4 expression showed lack of difference between control and fipronil treated cells. However, further studies are needed to clarify the issue.

We report the first in vivo and in vitro effects of fipronil on lungs and macrophage cells, respectively. The data show lung inflammation following both oral and intranasal treatments with fipronil and an increase in TLR4 positive cells in alveolar septa with intranasal treatment. The in vitro treatment with fipronil caused a concentration dependent reduction in the number of viable U937 macrophage cells but had no effect on the TLR4 or TLR9 expression. Taken together, these data suggest that exposure to fipronil induces lung inflammation and may increase its susceptibility for subsequent endotoxin exposure.
